# Association of heart rate variability with cardiorespiratory fitness and muscle strength in patients after hospitalization for COVID-19: An analytical cross-sectional study

**DOI:** 10.1016/j.clinsp.2024.100534

**Published:** 2024-11-19

**Authors:** Daniele Ferreira Rodrigues, Victor Ribeiro Neves, Ulisses Ramos Montarroyos, Washington José dos Santos, Isabelle Carolline Verissimo de Farias, Dário Celestino Sobral Filho

**Affiliations:** a Health Sciences Postgraduate Program at the Universidade de Pernambuco (UPE), Recife, PE, Brazil; b Hospital das Clínicas da Universidade Federal de Pernambuco (UFPE), Recife, PE, Brazil; c Functional Rehabilitation and Performance Postgraduate Program at the Universidade de Pernambuco (UPE), Recife, PE, Brazil; d Public Health Postgraduate Program, Universidade Estadual do Ceará (UECE), Fortaleza, CE, Brazil

**Keywords:** COVID-19, Heart Rate, Autonomic Nervous System, Walk Test, Muscle Strength

## Abstract

**Introduction:**

Patients with a moderate to severe clinical condition of COVID-19 who need hospitalization may have dysfunction in Cardiac Autonomic Control (CAC) and functional capacity.

**Objective:**

To assess the association of HRV with cardiorespiratory fitness and respiratory and peripheral muscle strength in patients after hospitalization for COVID-19.

**Method:**

Cross-sectional study with individuals > 18-years old, post-hospitalization for COVID-19, with a positive RT-PCR test. Data on clinical condition and hospital admission were collected, and parameters of respiratory and peripheral muscle strength and functional capacity were evaluated with the 6-Minute Walk Test (6MWT). CAC was assessed by analyzing (Heart Rate Variability) HRV in the time (SDNN, RMSSD) and frequency domains (HF, LF, HF/LF ratio). Pearson correlation was performed between HRV measures and functional parameters.

**Results:**

The results showed low HRV in study participants, with positive correlations between 6MWT and RMSSD and between SDNN and HF power and negative correlations between Heart Rate and the LF/HF ratio. On the other hand, respiratory and peripheral muscle strength correlated positively with parameters that represent sympathetic nervous system expression (LF nu and LF power) and negatively with the LF/HF ratio. However, changes in HRV parameters were not associated with disease severity.

**Conclusion:**

The dysautonomia of COVID-19 patients was correlated with functional sequelae, though not associated with disease severity parameters. There was low HRV, with low vagal expression, and imbalance in sympathetic/parasympathetic modulation in the study group.

## Highlights

• Respiratory and peripheral strength measurements are positively correlated with LF and the LF/HF ratio.
• Respiratory and peripheral strength measurements are negatively correlated with HF after COVID-19.
• Respiratory and peripheral muscle strength positively correlates with sympathetic nervous system parameters.
• Respiratory and peripheral muscle strength correlates negatively with parameters related to the parasympathetic nervous system.
• Cardiorespiratory fitness showed a positive correlation between the distance covered in the 6MWT.

## Introduction

COVID-19, a disease caused by the SARS-CoV-2 virus, despite some particularities such as high pathogenicity, resembles other previously experienced viral conditions.^1^ It has a spectrum of severity that ranges from asymptomatic to acute respiratory infection with high morbidity and mortality.^2^ Although it was initially identified as a respiratory infection, COVID-19 can also present acute and chronic manifestations in several other systems, such as neurological, pulmonary, cardiac, and gastrointestinal.^1^^,^^3^

Although COVID-19 has serious acute effects mainly related to severe respiratory failure, some studies have highlighted its long-term effects. Multisystem dysfunction that can persist for more than 12 weeks has been called long COVID or post-COVID-19 syndrome,^3^ which includes symptoms such as fatigue, muscle weakness, reduced tolerance to exercise with dyspnea and cough, malaise after exertion, chest pain, palpitations, and “brain fog”.^3^^,^^4^ Furthermore, the most seriously ill patients requiring hospitalization develop a series of complications that contribute to the functional limitations observed after discharge,^5^ especially if they overlap with post-Intensive Care (ICU) syndrome.^1^

Some authors have demonstrated the importance of evaluating these patients’ functional capacity. The 6MWT is a valid measure of exercise capacity for people with chronic lung disease and has proven to be important for monitoring post-COVID-19, correlating with the severity of the disease in the acute phase and with losses in the chronic phase.^6^

Besides the complications that may arise from the hospital stay, a growing body of evidence suggests an association between persistent post-COVID-19 symptoms and Cardiac Autonomic Control (CAC) dysfunction.^7^, ^8^, ^9^, ^10^ Some of these symptoms can be considered a clinical expression of cardiac dysautonomia related to COVID-19 and are present in postural orthostatic tachycardia syndrome and orthostatic hypotension.^9^^,^^10^ Post-COVID-19 patients had a 15 % prevalence of cardiac autonomic dysfunction, higher levels of inflammatory markers, and greater disease severity at the time of initial presentation.^10^

Heart Rate Variability (HRV) is a non-invasive method of assessing CAC. It evaluates interval oscillations between consecutive heartbeats (R-R intervals), which are influenced by the Autonomic Nervous System (ANS) on the sinus node. Reduced HRV may be associated with physiological and pathological conditions, and a low HRV is considered a sign of compromised ability to respond appropriately to stimuli coming from the ANS.^11^

To date, the literature relating COVID-19 to HRV is still scarce.^12^ Some studies have found lower HRV in patients who recovered from COVID-19,^13^, ^14^, ^15^ with worse parameters in those who had more severe forms of the disease.^10^ Worse autonomic modulation may also be associated with factors such as fatigue^13^ and shorter time since diagnosis.^14^

Despite emerging evidence, there is little information about the relationship between HRV and physical fitness in this population. Therefore, this study aims to evaluate the association between Heart Rate Variability (HRV), cardiorespiratory fitness and respiratory and peripheral muscle strength in individuals who were hospitalized for COVID-19. Additionally, the study aims to examine the correlation between HRV and hospitalization data, as well as disease severity. This secondary objective could provide information on whether there is a relationship between autonomic dysfunction reflected in HRV and the severity of COVID-19 disease experienced by patients.

The hypothesis suggests that cardiac dysautonomia, a condition characterized by dysfunction of the autonomic nervous system that affects heart rate regulation, may be a factor present in post-COVID-19 patients. By addressing these objectives, the study can contribute to a better understanding of the physiological implications of COVID-19 on the cardiovascular and autonomic nervous systems, as well as its potential long-term effects on physical fitness and health outcomes.

## Materials and methods

### Study design and setting

Analytical cross-sectional study carried out at the Post-ICU Outpatient Center of the Clinics Hospital of the Federal University of Pernambuco. The study was approved by the Research Ethics Committee of the HUOC/PROCAP of the University of Pernambuco under evaluation report n° 4448,602. All participants were duly informed about the study procedures and objectives; after agreeing, they signed an informed consent form.

The study was carried out at the Post-ICU Outpatient Clinic of Hospital de Clínicas, Pernambuco, Brazil. The Hospital das Clínicas was a regional reference for assistance to critically ill patients during the COVID-19 pandemic. The Post-ICU Outpatient Clinic was created to support patients after hospital discharge, it is made up of a multidisciplinary team and has the support of all hospital clinics for referrals and consultations.

### Participant recruitment and selection criteria

The study inclusion criteria were individuals over 18 years of age, with a history of hospitalization for COVID-19 confirmed by a positive PCR-RT test and admitted to the HC-UFPE post-intensive care outpatient clinic. The exclusion criteria are Individuals with a hospital stay of less than 48 h, who were unable to perform functional tests due to physical and/or cognitive limitations and who had limitation criteria for the assessment of CAC through HRV: complex cardiac arrhythmias, heart transplant, cardiac pacemaker, Atrioventricular Block (AVB) and progressive neuromuscular disease.

### Data collection

**Fig. 1:**
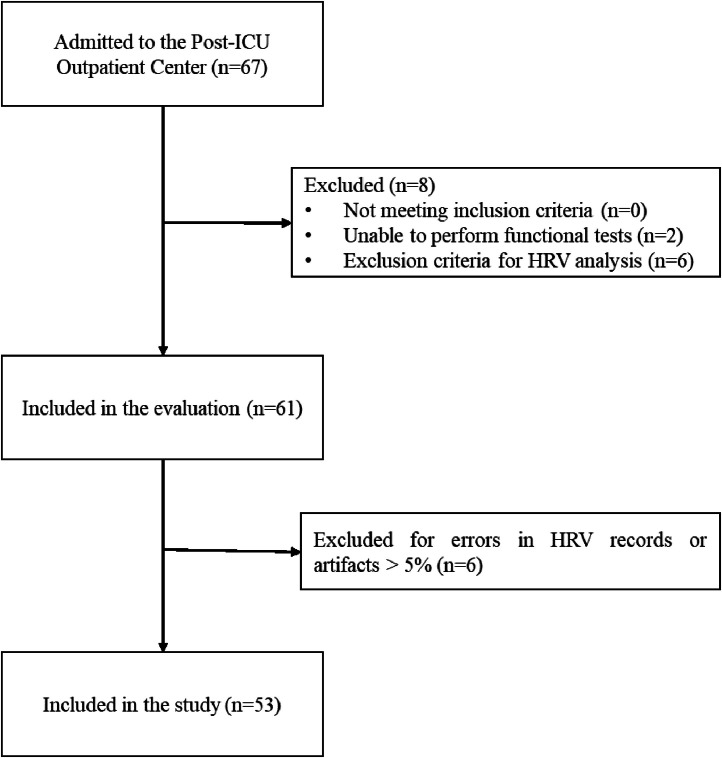
Flow diagram of participants.

Data collection began in September 2020, when the Post-ICU Outpatient Clinic was opened for patients’ post-hospitalization due to COVID-19, ending in November 2021 with a reduction in cases referred to this service. Sample size calculation was not performed, all patients admitted to the Post-ICU Outpatient Clinic were evaluated and those who met the inclusion criteria were included in the study (Fig. 1).

Trained physiotherapists using standardized protocols performed all assessments. Approximately 60 days after hospital discharge, patients were recruited for evaluation. Sociodemographic and clinical data were collected: sex, age, weight and height, pre-existing diseases and medications in use. Body Mass Index (BMI) was calculated by dividing weight by height, in meters, squared, and individuals with BMI > 30 were classified as obese.

Hospitalization data were obtained by consulting medical records or hospital discharge summaries. The following was recorded in days: hospitalization time, ICU need, ICU time, use of invasive mechanical ventilation, invasive mechanical ventilation time, use of tracheostomy, tracheostomy time, and need for hemodialysis during hospitalization.

### Response variables

CAC was assessed by analyzing HRV data. HRV was recorded with the Polar V800® heart rate monitor and Polar H10® sensor connected to a Polar Pro® elastic strap (Polar ElectroOy, Kempele, Finland). RR interval recordings followed the recommendations of the Task Force of the European Society of Cardiology and the North American Society of Pacing and Electrophysiology^16^ and the standardization procedure checklist regarding the use of collection methodology and data analysis.^17^ Each recording lasted 15 min, with the participant in a sitting position and at rest, and was transferred to the Polar FlowSync® program. Next, the RR intervals were exported to Kubios HRV Standard – version 3.3.1 (MATLAB®), in which the RR intervals were processed as a time series of 256 sequential beats, digitally filtered to eliminate artifacts, and the HRV indices were calculated. Records that had errors or artifacts >5 % were excluded. HRV parameters were used in linear methods in the frequency and time domains (Table 1).

**Table 1:** Parameters of the heart rate variability through linear methods (time and frequency domains).

Parameter	Unit	Description	Autonomic Reflection
HRV linear measures time domain			
SDNN	ms	The standard deviation of NN intervals	PNS and SNS activity
RMSSD	ms	Root mean square of successive RR interval differences	PNS activity
HRV linear measures frequency domain			
LF power	ms^2^	Absolute power of the low-frequency band (0.04–0.15 Hz)	Mixed PNS and SNS activity^a^
LF power	nu	Relative power of the low-frequency band (0.04–0.15 Hz) in normal units	Mixed PNS and SNS activity^a^
HF power	ms^2^	Absolute power of the high-frequency band (0.15–0.4 Hz)	PNS activity
HF power	nu	Relative power of the high-frequency band (0.15–0.4 Hz) in normal units	PNS activity
LF/HF	%	Ratio of LF-to-HF power	SNS-to-PNS balance^a^

PNS, Parasympathetic Nervous System; SNS, Sympathetic Nervous System.

a Further research is needed on autonomic reflection. Source: Shaffer, Ginsberg, 2017; Dobbs et al., 2019.^11^^,^^20^

Respiratory muscle strength was assessed by measuring the Maximum Inspiratory Pressure (MIP) and Maximum Expiratory Pressure (MEP) with a digital respiratory pressure meter MDV 300 (Globalmed®) and following the criteria of the American Thoracic Society and European Respiratory Society (ATS/ERS).^18^ Predicted values for sex and age were calculated.^19^

The Medical Research Council (MRC) and Palmar Grip Strength (PGS) were applied to assess peripheral muscle strength. The MRC identifies the muscle's ability to contract against resistance manually imposed by the evaluator with scores ranging from 0 (zero) (complete muscular paralysis) to 5 (normal movement against great resistance) for each muscle group (shoulder abductor, elbow flexor, and wrist extensor, in the upper limbs; hip flexor, knee extensor, and dorsiflexors, in the lower limbs), resulting in a maximum score of 60 points.^21^ Dynamometry measures PGS in kilogram-force (kgf), based on the spring deformation (CAMRY Dynamometer, model EH101, Guangdong, China). It was performed on the dominant limb, in the sitting position, arm parallel to the trunk, shoulder in neutral position, and elbow at 90 °C. The highest measurement out of three consecutive repetitions was chosen.

Cardiorespiratory fitness was assessed based on the distance covered in the 6-minute Walk Test (6MWT) in a 30-meter-long corridor, according to ATS/ERS guidelines.^22^ Standardized instructions and verbal encouragement were provided to participants every minute. The distance covered in the 6MWT was recorded in meters, and the predicted values were calculated based on the reference equation for the Brazilian population.^23^

### Statistical analysis

Statistical analysis was performed in STATA SE software version 14 (STATA SE 14, StataCorp LP, College Station, TX, USA). The description of clinical characteristics was presented in the frequency distribution, and continuous variables were presented as the mean, standard deviation, median, Q1, and Q3. The standard deviation or median, depending on their normality, is verified with the Kolmogorov-Smirnov test.

Initially, a Mann-Whitney association test was performed to test the association between sociodemographic and clinical data and HRV indices. This test was used to define possible confounding variables, with the sex variable being the most associated with HRV (Supplementary Material). The association between HRV indices and COVID-19 severity parameters was then tested using multiple linear regression, which estimated the regression coefficient of the variability measures adjusted for the patient's sex and age. The correlation between HRV and functional parameters (functional capacity and muscle strength) was analyzed using Pearson's correlation coefficient estimates. For both tests, the logarithmic transformation was applied with the aim of normalizing variables that did not present a normal distribution and allowing the use of parametric tests, improving the quality of the statistical model used. The statistical significance of the study was set at 5 % (p < 0.05).

## Results

During the study period, 67 post-COVID-19 patients were admitted to the Post-ICU Outpatient Clinic of the Hospital de Clínicas of the Federal University of Pernambuco, two were excluded due to physical or cognitive inability to perform the functional tests and six were exclusion criteria for HRV analysis. Of the 61 included in the study, after HRV collection, 6 were excluded due to recording errors or artifacts >5 %. Resulting in a final sample of 53 individuals for data analysis.

Table 2 presents the participants’ clinical data and hospital stays. They had a mean age of 52.2 years (± 11.4), and a BMI median of 31.4 (28.7–35.3), with obesity and hypertension being the most prevalent morbidities. The median hospitalization time was 21 days (11‒42), and 34 patients (64.1 %) needed admission to the ICU.

**Table 2:** Clinical characteristics and hospital stay data of patients recovered from severe acute respiratory syndrome due to COVID-19 (n = 53).

Characteristics	n = 53
**Sex, males**	24 (45.3 %)
**Age, years**^b^	52.2 ± 11.4
**Weight, kg**^b^	87.3 ± 21.2 kg
**BMI, kg/m^2^**^c^	31.4 (28.7 – 35.3)
**Health-related factors**^a^	
Obesity^d^	33 (62.3 %)
Smoking	14 (26.9 %)
Alcohol consumption	10 (19.2 %)
SAH	30 (56.6 %)
Type 2 diabetes mellitus	15 (28.3 %)
CRD	4 (7.5 %)
Asthma	9 (17.0 %)
**Medications in use**^a^	
Diuretics	8 (15.1 %)
Antihypertensive drugs	32 (60.4 %)
Anticoagulants	12 (22.6 %)
Oral antidiabetics	13 (24.5 %)
Bronchodilators	7 (13.2 %)
Analgesics	4 (7.5 %)
**Hospital stay data**	
Hospital length of stay, days^c^	21 (11 – 42)
ICU stay	34 (64.1 %)
ICU length of stay, days^c^	12.5 (5 – 22)
MV use	20 (37.7 %)
Time of MV use, days^c^	19 (10 – 30)
Tracheostomy	7 (13.7 %)
Time of tracheostomy, days^c^	15 (6 – 26)
Hemodialysis during the hospital stay	9 (18.4 %)

a Non-excluding categories.

b Values are expressed as mean ± standard deviation.

c Data are presented as median (first quartile – third quartile).

d Considering BMI ≥ 30 kg/m^2^. BMI, Body Mass Index; COPD, Chronic Obstructive Pulmonary Disease; CRD, Chronic Renal Disease; ICU, Intensive Care Unit; MV, Invasive Mechanical Ventilation; SAH, Systemic Arterial Hypertension.

Functional assessment and HRV data are described in Table 3, which also presents the percentages of reference values calculated with predictive equations. There were deficits in respiratory muscle strength (MIP: 75.5±26.8 % of the predicted value; MEP: 80.3±26.5 % of the predicted value) and low functional capacity in 6MWT (71.9±22.6 % of the predicted value).

**Table 3:** Distribution of the measures of cardiorespiratory fitness, respiratory and peripheral muscle strength, and heart rate variability of patients recovered from severe acute respiratory syndrome due to COVID-19 (n = 53).

Measures	Mean ± standard deviation
**Respiratory muscle strength**	
MIP (cm H_2_O)	74.8 ± 31.1
% Predicted MIP	75.5 ± 26.8
MEP (cm H_2_O)	82.4 ± 32.6
% Predicted MEP	80.3 ± 26.5
**Peripheral muscle strength**	
MRC	55.1 ± 6.1
PGS	27.2 ± 12.1
**Cardiorespiratory fitness**	
6MWT, meters	369 ± 122
Predicted 6MWT (%)	71.9 ± 22.6
**Heart rate variability**	
Mean heart rate (bpm)	82.3 ± 12.4
RR interval (ms)	746 ± 118
SDNN (ms)^a^	16 (10 – 23.1)
RMSSD	12.5 (8 – 22)
Total LF power (ms^2^)^a^	106 (43 – 257)
LF (nu)	65.6 ± 21.9
Total HF power (ms^2^)^a^	52 (13 – 144)
HF (nu)	34.2 ± 21.9
LF/HF ratio^a^	2.5 (1.0 – 5.5)

a Data are presented as median (first quartile – third quartile) 6MWT, 6-Minute Walk Test; HF, High Frequency; LF, Low Frequency; LF/HF, Low-Frequency to High-Frequency Ratio; MEP, Maximum Expiratory Pressure; MIP, Maximum Inspiratory Pressure; MRC, Medical Research Council; PGS, Palmar Grip Strength; RR interval, Mean of RR intervals; RMSSD, Root Mean Square of Successive Differences between adjacent normal RR intervals; SDNN, Standard Deviation of the mean Normal RR intervals.

Table 4 presents the results of the linear regression, adjusted for sex and age, between HRV indices and hospitalization data related to the severity of COVID-19 (e.g., length of stay, length of ICU stay, etc.). No statistical association was observed between the variables analyzed.

**Table 4:** Association between heart rate variability and parameters related to the severity of COVID-19 in patients recovered from severe acute respiratory syndrome due to COVID-19 (n = 53).

Measures	Mean HR β coef (p-value)	SDNN^b^ β coef (p-value)	RMSSD^b^ β coef (p-value)	LF (nu) β coef (p-value)	HF (nu) β coef (p-value)	LF/HF ratio^b^ β coef (p-value)
**Length of hospital stay (days)**	-0.015 (0.861)	-0.004 (0.351)	-0.003 (0.556)	0.010 (0.943)	-0.009 (0.948)	-0003 (0.961)
**ICU admission**						
No	Ref	Ref	Ref	Ref	Ref	Ref
Yes	4.45 (0.236)	-0.273 (0.120)	-0.302 (0.157)	7.73 (0.205)	-7.70 (0.206)	0.394 (0.221)
**Length of ICU stay (days)**	0.060 (0.629)	-0.007 (0.235)	-0.004 (0.568)	-0.012 (0.951)	0.014 (0.944)	-0.002 (0.853)
**MV use**						
No	Ref	Ref	Ref	Ref	Ref	Ref
Yes	-0.43 (0.909)	-0.116 (0.506)	-0.008 (0.971)	-4.46 (0.459)	4.44 (0.461)	-0.299 (0.348)
**Length of MV use (days)**	-0.221 (0.299)	-0.001 (0.942)	0.006 (0.639)	-0.237 (0.495)	0.238 (0.492)	-0.014 (0.461)
**Tracheostomized**						
No	Ref	Ref	Ref	Ref	Ref	Ref
Yes	0.52 (0.921)	-0.276 (0.236)	-0.173 (0.550)	-6.27 (0.463)	6.35 (0.457)	-0.320 (0.480)
**Duration of tracheostomy (days)**	0.187 (0.393)	-0.011 (0.276)	-0.009 (0.450)	0.030 (0.935)	-0.027 (0.940)	0.001 (0.963)
**Hemodialysis during hospital stay**						
No	Ref	Ref	Ref	Ref	Ref	Ref
Yes	-2.95 (0.533)	-0.056 (0.796)	0.057 (0.829)	-3.08 (0.690)	3.12 (0.686)	0.008 (0.985)

^a^ Linear regression coefficients adjusted for sex and age;

b Transformation applied with logarithmic function to normalize the distribution. ICU, Intensive Care Unit; MV, Invasive Mechanical Ventilation; HF, High Frequency; HR, Heart Rate; LF, Low Frequency; LF/HF, Low-Frequency to High-Frequency ratio; RR interval, mean of RR intervals; RMSSD, Root Mean Square of Successive Differences between adjacent normal RR intervals; SDNN, Standard Deviation of the mean Normal RR intervals.

Table 5 shows the correlations between HRV indices and functional assessment data. Respiratory and peripheral strength measures (MIP, MEP, MRC, and PGS) were positively correlated with LF and the LF/HF ratio and negatively correlated with HF. Functional capacity, assessed in 6MWT, was positively correlated with SDNN (*r* = 0.342, p = 0.016), RMSSD (*r* = 0.432, p = 0.002), and HF (*r* = 0.375, p = 0.008) and negatively correlated with the LF/HF Ratio (*r* = -0.288, p = 0.045).

**Table 5:** Correlation between the heart rate variability and functional parameters of patients after hospitalization due to COVID-19.

Heart Rate Variability	Respiratory muscle strength		Peripheral muscle strength		Cardiorespiratory fitness
	MIP	MEP	MRC	PGS	6MWT^b^
	r (p-value)	r (p-value)	r (p-value)	r (p-value)	r (p-value)
Mean heart rate (bpm)	0.211 (0.136)	0.097 (0.492)	-0.091 (0.529)	0.118 (0.420)	**-0.361 (0.011)**
SDNN (ms)^a^	-0.013 (0.928)	-0.101 (0.474)	0.165 (0.252)	0.011 (0.941)	**0.342 (0.016)**
RMSSD^a^	-0.158 (0.264)	-0.237 (0.091)	0.050 (0.732)	-0.133 (0.361)	**0.432 (0.002)**
LF power (ms^2^)^a^	**0.344 (0.012)**	0.249 (0.075)	**0.364 (0.009)**	**0.319 (0.026)**	-0.240 (0.096)
LF (nu)	**0.389 (0.004)**	**0.322 (0.020)**	**0.356 (0.011)**	**0.322 (0.024)**	-0.261 (0.071)
HF power (ms^2^)^a^	-0.238 (0.089)	**-0.291 (0.036)**	-0.034 (0.834)	-0.189 (0.192)	**0.375 (0.008)**
HF (nu)	**-0.390 (0.004)**	**-0.322 (0.020)**	**-0.356 (0.011)**	**-0.322 (0.024)**	0.258 (0.073)
LF/HF ratio^a^	**0.388 (0.004)**	**0.352 (0.010)**	**0.355 (0.017)**	**0.348 (0.014)**	**-0.288 (0.045)**

a Transformation applied with logarithmic function to normalize the distribution.

b Percentage used in relation to the calculated values through predictive equations. 6MWT, 6-Minute Walk Test; HF, High Frequency; HR, Heart Rate; LF, Low Frequency; LF/HF, Low-Frequency to High-Frequency ratio; MEP, Maximum Expiratory Pressure; MIP, Maximum Inspiratory Pressure; MRC, Medical Research Council; PGS, Palmar grip strength; RR interval, Mean of RR Intervals; RMSSD, Root Mean Square of Successive Differences between adjacent normal RR intervals; SDNN, Standard Deviation of the mean Normal RR intervals.

## Discussion

This study was carried out with patients who presented moderate to severe respiratory failure due to COVID-19, requiring hospital admission. There was a prevalence of 64 % of ICU admissions and 37 % of invasive mechanical ventilation, with prolonged hospitalization. The profile of the patients evaluated had a mean age of 52.2 years (±11.4), with a high BMI and a prevalence of obesity of 62.3 %. This profile has already been described in the literature as presenting an increased risk of severity for COVID-19.^24^

The functional assessment data identified low respiratory muscle strength in the individuals evaluated, with 75.5 % ± 26.8 and 80.3 % ± 26.5 of the predicted MIP and MEP respectively.^19^ Cardiorespiratory fitness was also below predicted according to the predictive equations, with a distance in the 6MWT of 71.9 % ± 22.6 of predicted[23] (Table 2). It is known that patients after recovery from the acute phase of COVID-19 may present clinical and functional sequelae that last for several months, either as a result of the viral infection itself or as a result of the critical illness.^3^^,^^4^

The study of autonomic heart rate control through HRV is widely used in various clinical situations, including ICU patients. This study used linear HRV indices in the time and frequency domains to evaluate cardiac autonomic control. HRV indices were weakly and moderately correlated with functional data (muscle strength and cardiorespiratory fitness) (Table 5), though not associated with variables relating to the severity of the disease or hospital admission data, such as length of stay and need for invasive MV (Table 4).

### Heart rate variability at rest

In general, this study found low parameters, in all HRV indices, in the patients evaluated when compared with short-term reference values for the Brazilian population.^31^ Global impairment of HRV was observed, with median values of SDNN = 12 (8–22), RMSSD = 16 (10–23.1) and LF/HF ratio = 2.5 (1.0–5.5) (Table 3). These findings demonstrate low sympathetic and parasympathetic expression in the patients studied with mild SNS predominance, which corroborates a recent study in patients 20 weeks after mild COVID-19 infection, without hospital admission, which found that they had significantly lower values of HRV than healthy controls in the time (SDNN, RMSSD) and frequency domains (LF, HF, LF/HF Ratio).^25^

The relationship between HRV and COVID-19 has already been studied and described in the literature. A study with critically ill patients observed low HRV parameters in patients using MV, with this dysautonomia being more pronounced in critically ill patients with COVID-19 (Silva, 2023). Some factors may explain the dysautonomia found in these patients, it may be ascribed to direct damage due to the SARS-CoV-2 virus,^7^ factors triggered by viral infection (e.g., hypoxia, exacerbated inflammatory process, and imbalanced renin angiotensin aldosterone system), or exacerbated activation of the sympathetic ANS by a series of mechanisms that involve immune-mediated reaction and inflammatory response and create a vicious circle that worsens the clinical condition.^26^

Nevertheless, there was a predominance of the sympathetic ANS (higher mean LF/HF ratio) (Table 3), which was also found in a study in patients with consistent post-COVID-19 symptoms. It observed reduced vagal expression and predominance of the sympathetic nervous system with lower HF values and higher mean LF/HF ratio than in healthy controls.^27^ This indicates worse autonomic modulation in these patients with persistence over time.

### HRV and 6-minute walk test

In this study, the 6MWT correlated positively with the SDNN, RMSSD and HF indices (*r* = 0.342, p = 0.016, *r* = 0.432, p = 0.002; *r* = 0.375, p = 0.008, respectively) and negatively with HR (*r* = -0.361, p = 0.011). It is worth noting that the resting Heart Rate (HR) is modulated mainly by the parasympathetic nervous system, through the vagus nerve.^28^ SDNN is a measure related to global modulation or autonomic balance, while RMSSD and HF represent vagal or parasympathetic autonomic modulation.^29^^,^^30^ These findings demonstrate better global autonomic control, observed through the SDNN, in patients with improved cardiorespiratory fitness, assessed by the 6 MWT.

Furthermore, it was also observed that the LF/HF ratio (which represents sympathovagal balance, i.e., higher values may indicate imbalance with a predominance of sympathetic ANS), was negatively correlated with TC6 (*r* = -0.288, p = 0.045), corroborating the finding that better cardiorespiratory fitness is related to more efficient cardiac autonomic modulation.

There is evidence that regular physical exercise promotes beneficial cardiac autonomic adjustments and that individuals with moderate to high cardiovascular fitness have a low risk of mortality from cardiovascular diseases.^31^ However, little evidence of the association between HRV and cardiorespiratory fitness was found and this remains controversial. No association was observed between HRV measurements and cardiorespiratory fitness in a study carried out with healthy individuals,^32^ as well as with patients with advanced heart failure.^33^ On the other hand, a systematic review concluded that moderate to vigorous physical activity is positively associated with RMSSD. This study also found an association between cardiorespiratory fitness and HRV, however, this relationship is less well-established.^34^

### HRV and Muscle strength

There was an opposite HRV behavior regarding respiratory and peripheral muscle strength, as MIP, MEP, MRC, and PGS correlated positively with the LF/HF ratio and LF (which indicates the modulation of sympathetic ANS) and negatively with HF (which represents vagal activity)[29] (Table 5). The results of the present study show that individuals with a lower capacity to generate peripheral muscular strength have an autonomic imbalance and lower sympathetic nervous system activity.

The literature has previously described this association between breathing, respiratory muscle strength, and HRV in patients with chronic obstructive pulmonary disease[35] and heart failure.^36^ It has been found that individuals with respiratory muscle weakness have abnormal CAC, characterized by reduced parasympathetic response.^35^ These results can be explained by the close relationship between breathing and cardiac autonomic modulation.^11^

A study that evaluated the relationship between respiratory muscle strength and cardiac autonomic modulation in patients with chronic heart failure observed that respiratory muscle weakness was associated with a more pronounced reduction in vagal tone in the sinus node.^36^ The shallow breathing and consequently the low tidal volume, caused by respiratory muscle weakness, leads to an early activation of the ergoreceptor responsible for the rapid response of the ANS, thus causing hyperactivation of the SNS.^36^

The relationship between peripheral muscle strength and HRV is still little explored in the literature. The gain in muscle strength after a strength training protocol has been accompanied by an improvement in cardiac autonomic modulation in patients with COPD, with a significant increase in the sympathetic and parasympathetic components of the ANS, represented by SDNN, LF and HF.^37^ Another study that evaluated the relationship between HRV and sarcopenia in the elderly found a positive correlation between muscle mass and SDNN, LF and HF, but did not observe significant differences in relation to Hand Grip Strength.^38^

### Limitations

Some considerations and limitations must be mentioned to properly interpret the findings of this study. It was carried out on a specific group of patients after hospitalization for COVID-19, these patients generally present important functional deficits due not only to severe COVID-19 infection, but also to the consequences of critical illness and prolonged hospital stay. Therefore, these results cannot be extrapolated to patients who had mild or asymptomatic forms of the disease.

Furthermore, HRV fluctuates throughout the day and varies according to different activities and factors such as diet, stress and sleep patterns, among others than those included in the preliminary analysis. The evaluation of these variables was not carried out in the present study, which may have impacted the findings. Future studies should include other control variables in their analyses. Finally, this study also has limitations inherent to the cross-sectional design, as it did not evaluate how HRV changes in these patients over time.

## Conclusions

Although much progress has already been made in understanding and managing post-COVID-19 syndrome, it continues to affect a considerable portion of the population affected by the disease. In general, low HRV was observed, with low vagal expression and imbalance in sympathetic/parasympathetic modulation in the study patients.

The dysautonomia related to COVID-19 observed in this study is correlated with functional sequelae and is not associated with disease severity parameters. Respiratory and peripheral muscle strength correlated positively with parameters that represent expression of the sympathetic nervous system and negatively with those related to the parasympathetic nervous system. On the other hand, functional capacity had the opposite behavior, with a positive correlation between the distance covered in the 6MWT and the parameters that best represent parasympathetic cardiac autonomic modulation or vagal modulation. Further research is needed with other methodological models and populations to understand these facts better and develop therapeutic interventions to address this debilitating syndrome.

## Data availability statement

The datasets used and/or analyzed during the current study are available from the corresponding author upon reasonable request.

## Financial support

This research did not receive any specific grants from public, commercial, or non-profit sector funding agencies.

## Authors' contributions

All authors read and agreed with the published version of the manuscript. (DFR, VRN, URM, and DCSF) participated in the conceptualization, project, and data analysis and interpretation; (DFR, WJS, and ICVF) contributed to article writing, (VRN and DCSF) made a critical review of important intellectual content and contributed to the approval of the final version for publication.

## Declaration of competing interest

The authors declare no conflicts of interest.

## Appendix Supplementary materials

Image, application 1
