# Supplementary material for: Association of heart rate variability with cardiorespiratory fitness and muscle strength in patients after hospitalization for COVID-19: An analytical cross-sectional study
Source: Clinics (Sao Paulo). 2024 Nov 19;79:100534. doi: 10.1016/j.clinsp.2024.100534 (PMC11617900; doi:10.1016/j.clinsp.2024.100534)
Supplement: Supplementary file 1 [file mmc1.docx]

CLINICS-D-23-00772_Supplementary Material

**Supplementary Material Table 1** Association between heart rate variability and sociodemographic and clinical data in patients recovered from severe acute respiratory syndrome due to COVID-19 (n = 53).

| **Heart Rate Variability** | **Sexo** | **Age^a,b^** | **Obesity** | **SAH** | **DM2** | **Asthma** | **DRC** |
| --- | --- | --- | --- | --- | --- | --- | --- |
| Mean heart rate (bpm) | 0.692 | 0.662 | 0.447 | 0.905 | 0.352 | 0.505 | 0.703 |
| SDNN (ms) | 0.606 | **0.039** | 0.131 | **0.030** | 0.526 | 0.171 | 0.261 |
| RMSSD | **0.035** | 0.184 | 0.341 | 0.119 | 0.492 | 0.271 | 0.292 |
| LF power (ms^2^) | 0.580 | 0.054 | 0.104 | 0.055 | 0.608 | 0.195 | 0.182 |
| LF (nu) | **0.001** | 0.995 | 0.840 | 0.912 | 0.650 | 0.536 | 0.475 |
| HF power (ms^2^) | **0.033** | 0.151 | 0.361 | 0.140 | 0.554 | 0.136 | 0.377 |
| HF (nu) | **0.001** | 0.997 | 0.832 | 0.905 | 0.650 | 0.552 | 0.475 |
| LF/HF ratio | **0.001** | 0.998 | 0.832 | 0.897 | 0.650 | 0.536 | 0.475 |

^a^ Variable categorized 20–39-years old, 40–59-years, ≥60-years.

^b^ Kruskal-wallis test performed.
